# Retrospective Analysis of Disparities in Timing of Prehospital and Emergency Department Pain Management by Sex and Age

**DOI:** 10.5811/westjem.47072

**Published:** 2025-12-19

**Authors:** Douglas Moss, Natalie Boorjian, Aidan Mullan, Sarayna S. McGuire, John Anderson, Neha P. Raukar

**Affiliations:** * University of Chicago, Department of Medicine, Section of Emergency Medicine, Chicago, Illinois; † Emory University, Atlanta, Georgia; ‡ Alix School of Medicine, Mayo Clinic, Department of Quantitative Health Sciences, Division of Clinical Trials and Biostatistics, Rochester, Minnesota; § Mayo Clinic, Department of Emergency Medicine, Rochester, Minnesota

## Abstract

**Introduction:**

Acute long bone fractures, such as femur and humerus fractures, frequently lead to emergency department (ED) visits and require timely pain management. However, disparities in analgesia administration persist across age and sex. This study investigates how these intersecting patient characteristics affect the timing and receipt of analgesia in both prehospital and ED settings.

**Methods:**

We conducted a retrospective cohort study of adults (≥ 18 years of age) presenting to a Level I trauma center ED in 2022 with femur or humerus fractures. Demographics, analgesia timing, and receipt in both prehospital and ED settings were extracted from medical records. Our analysis included all forms of initial analgesic administration, including both narcotic and non-narcotic medications. We further categorized treatments to distinguish between any analgesia and narcotic analgesia. Multivariable Poisson and logistic regression models were used to assess disparities, adjusting for triage acuity, arrival method, initial pain score, and prehospital analgesia.

**Results:**

Among 553 patients, 75% were ≥ 65 of age and 63% were female. Older adults experienced significantly longer delays to ED analgesia compared to younger adults (median 81 vs 44 minutes; +54.9% adjusted delay; *P* < .001) and were less likely to receive prehospital analgesia (44% vs 66%; odds ratio 2.52; *P* < .001). Sex-based disparities were also evident: females waited longer than males for ED analgesia (median 76 vs 57 minutes; +12.9% adjusted delay; *P* < .001). Among those who received prehospital analgesia, females waited 43% longer than males for subsequent ED pain treatment (median 72 vs 30 minutes; *P* <.001).

**Conclusion:**

Age and sex disparities exist in both prehospital and ED pain management for long bone fractures. Older adults were less likely to receive prehospital analgesia and experienced prolonged delays in the ED. Female patients had longer ED wait times for analgesia, especially following prehospital treatment administered by emergency medical services responders.

## INTRODUCTION

Acute fractures are a common reason for emergency department (ED) evaluation, and managing these injuries requires balancing timely pain control with appropriate fracture care. However, pain management in the ED is often delayed and undertreated,^1^–^2^ with variations influenced by both patient and clinician factors.^3^–^9^ Clinicians may hesitate to prescribe opioids to younger patients due to concerns about addiction and misuse,^10^ while prescribing pain medication for older adults (> 65 years of age) is often complex due to multimorbidity, polypharmacy, altered drug metabolism, and the risk of opioid-induced delirium.^11^–^12^ These factors contribute to oligoanalgesia in this population, impacting their quality of life.

Disparities in analgesia delivery have been documented across patient characteristics, including age, sex, and ethnicity.^3^–^5^,^7^–^8^ Sex-based differences, particularly in the management of chronic musculoskeletal pain, are well-documented, with women often receiving less effective pain control.^13^ Older adults are disproportionately affected by osteoporotic fractures, which occur earlier in women due to their earlier onset of bone loss.^14^ In this study we investigated sex and age disparities in the delivery and timing of analgesia for patients with acute long bone fractures, focusing on femur and humerus fractures. These fractures, hallmark injuries of osteoporosis, often carry significant morbidity and demand prompt and effective pain management. The intersection of sex- and age-related differences in acute fracture pain management, both in the prehospital and the hospital setting, particularly in geriatric patients, remains unclear.

## METHODS

This retrospective cohort study included patients ≥ 18 years of age who presented to a single, Level 1 trauma center ED between January 1–December 31, 2022, with a final diagnosis of a femur or humerus fracture. The ED has an annual volume of approximately 80,000 visits. Patients were identified through diagnostic codes in the electronic health record (Epic Systems Corporation, Verona, WI), and all eligible patients during the study period were included. We used a consecutive sampling method to ensure inclusion of all eligible cases. Data were abstracted using standardized data collection forms. Abstracted variables included patient age, biologic sex, fracture type, means of arrival (emergency medical. services [EMS] vs private vehicle), whether pain medication was administered in the prehospital and ED settings, and time from ED arrival to analgesia administration.

We abstracted analgesic administration as two binary variables: any analgesia (yes/no), and any opioid (yes/no). We did not collect data on specific nonopioid analgesics (eg, acetaminophen, non-steroidal anti-inflammatory drugs, ketamine). For patients transported by EMS, prehospital medication administration data were extracted from the EMS database. Due to frequent inaccuracies in recorded administration times, only the presence or absence of prehospital analgesia was recorded. We did not include nerve blocks and non-pharmacologic treatments in our analysis as they were not used as initial interventions in either the prehospital or ED setting. In contrast, both administration and timing of analgesia were reliably recorded in the ED.

One author SM with extensive experience with the EMS database trained a second abstractor. Abstractor performance was monitored throughout data collection. To minimize bias, the abstractor collecting prehospital data was blinded to the study hypothesis until after data collection was complete. We evaluated interobserver reliability on a subset of cases, with complete agreement (κ = 1.0) observed across all abstracted variables. Missing data were infrequent and are described where applicable in the results. No imputation was performed; we conducted analyses using available case data. **Regarding triage levels**, patients with similar injuries may have been assigned different Emergency Severity Index (ESI) levels based on presenting vital signs, comorbidities, or initial pain severity. To account for this, we included ESI level as an adjustment variable in all regression models, and Table 1 was updated to ensure internal consistency across comparisons.

**Population Health Research Capsule**

What do we already know about this issue?

*Disparities in emergency pain management by age and sex exist, but their interaction across prehospital and emergency department settings is poorly understood*.

What was the research question?

*Do age and sex impact timing and likelihood of analgesia in prehospital and emergency care for long bone fractures?*

What was the major finding of the study?

*Older adults experienced significantly longer delays to ED analgesia compared to younger adults (81 vs 44 min; 54.9% longer; P < .001), as did females (76 vs 57 min; 43% longer; P < .01)*.

How does this improve population health?

*Findings highlight vulnerable groups—older adults and females—at risk for delayed pain care, informing targeted, equitable interventions*.

This study adhered to all 12 recommended methodological elements for retrospective chart review as outlined by Worster and Bledsoe,^15^ including clearly defined case selection criteria, standardized variable definitions, abstractor training and monitoring, blinding to the study hypothesis, use of abstraction forms, assessment of interobserver reliability, and appropriate handling of missing data. The Institutional Review Board (IRB) deemed the study exempt, and consent was waived by the IRB.

## DATA ANALYSIS

We divided patients into two age groups: adults (18–64 years) and older adults (≥ 65 years of age). Older adults were further stratified into 65–84 years and ≥ 85 years of age for sensitivity analyses. Sex was categorized as male or female based on hospital records.

The primary outcome of interest was the time from patient arrival in the ED to analgesia administration. We analyzed time to analgesia administration using multiple Poisson regression, with adjustments for triage ESI, means of arrival, prehospital analgesia, and initial pain score. Results were reported as adjusted percent differences (APD) with 95% CI. We conducted a secondary analysis to evaluate whether patients arriving at the ED via EMS received prehospital analgesia. Receipt of prehospital analgesia was compared between age groups and sex using univariable logistic regression. We report results with odds ratios (OR) and 95% CI.

## RESULTS

### Cohort Characteristics

A total of 553 patients with a femur or humerus fracture were included in this study; 414 (74.9%) were older adults (≥ 65 years of age), and 139 (25.1%) were adults18–64 years (Table 1). We collected initial pain scores for all patients and included those scores in our adjusted models. Female patients comprised 63.1% (n = 349) of the fracture patients while males accounted for 36.2% (n = 200).

### Age Analysis

Older adults experienced significantly longer wait times for pain medication after arriving in the ED compared to adults ≤ 64 years of age, with a median time of 81 minutes (IQR 41–144; Table 2) vs 44 minutes (IQR 19–88) (APD +54.9%, 95% CI 50.9–59.1%, *P* < .001; Table 3) after adjusting for triage ESI, first pain score in the ED, means of arrival, and prehospital analgesia.

In the prehospital setting, adults ≤ 64 years of age (66.3%) were more than twice as likely to receive analgesia compared to older adults ≥ 65 (43.8%, OR 2.52, 95% CI, 1.53–4.14, *P* < .001), and older adults 65–84 years of age were significantly more likely to receive prehospital analgesia compared to those aged ≥ 85 years (OR 1.72, 95% CI, 1.09–2.70, *P* = .02). Upon arrival to the ED via EMS, older adults who did receive prehospital pain medication experienced a significantly longer wait for additional pain management in the ED compared to younger adults (median 68 vs 30 minutes, APD +77.2%, 95% CI, 68.8–86.0%, *P* < .001), even when adjusting for initial pain score and triage ESI. Older adults arriving to the ED via private vehicle experienced significantly longer delays in receiving pain medication compared to adults ≤ 64 years of age, with an adjusted difference of approximately 33% after accounting for triage ESI and pain scores (median 101 vs 70 minutes, APD +32.8%, 95% CI, 15.7–52.4%, *P* < .001).

### Sex Disparities

Overall, female patients experienced longer delays in analgesia administration than males in the ED, with a median wait time of 76 minutes (IQR 41–135) vs 57 minutes (24–118). After accounting for means of arrival, prehospital analgesia, triage ESI level, and first pain score in the ED, females had a 13% longer wait for pain medication compared to males (APD +12.9%, 95% CI, 10.6–15.3%, *P* < 0.001). Among younger adults there was no difference in time to pain medication between females and males (median 49 vs 39 minutes; APD +1.0%, 95% CI, −3.7% to +5.9%, *P* = .68). However, older adult females waited significantly longer for pain medication compared to older adult males (median 83 vs 70 minutes; APD +5.0%, 95% CI, 2.5–7.5%, *P* < .001).

No sex differences were observed in prehospital analgesia administration (49.2% for females vs 47.7% for males, OR 1.06, 95% CI, 0.71–1.58, *P* = .76). However, among EMS-arriving patients who received prehospital analgesia, females waited 43% longer for additional pain medication in the ED compared to males (median 72 vs 30 minutes, APD +43.0%, 95%, CI 7.2–49.0%, *P* < .001). This difference in time to pain medication among EMS-arriving patients was significant among both age groups; adult females waited 59% longer than adult males (median 51 vs 21 minutes; APD +59.0%, 95% CI, 45.3–73.9%, *P* < .001) whereas older adult females waited 28% longer than older adult males (median 74 vs 43 minutes; APD +27.7%, 95% CI, 14.8–42.0%, *P* < .001).

## DISCUSSION

This analysis demonstrates clear age- and sex-based disparities in the timeliness of analgesia administration for patients with acute femur and humerus fractures across the prehospital and ED settings. Older adults, especially those aged ≥85 years, experienced significant delays in receiving analgesia in both prehospital and ED settings, even after adjustment for triage ESI, initial ED pain score, means of arrival, and prehospital analgesia. Female patients, regardless of age, have historically experienced delays in pain management, with the disparities being most pronounced in geriatric females > 65 years of age.^16^, ^17^ This finding aligns with prior research suggesting oligoanalgesia in women, possibly due to biases in pain perception or assumptions about their pain tolerance.^16^,^17^

Although our data suggest that sex disparities may be narrowing in the adult cohort, any inference of “progress” should be made cautiously, as this was a singlecenter, retrospective, cohort study. In the non-EMS cohort, there was no significant difference in pain medication administration or wait times between females and males (median 86 vs 96 minutes, *P* = .38). Additionally, while female adults waited less time than male adults (median 67 vs 82 minutes, *P* < .001), older females and males (> 65) had nearly identical wait times (median 101 vs 99 minutes, *P* = .09).

Because our primary aim was to examine timeliness of analgesic administration, rather than the adequacy of pain control, we did not include patient-reported relief measures. Timely analgesic administration for long bone fractures was a previously reported metric for the Joint Commission and a Centers for Medicare and Medicaid Services quality measure in 2013, highlighting the importance of prompt and equitable pain control. Although not a current Joint Commission metric, pain management remains central to its standards. These delays are particularly concerning given that femur and humerus fractures are hallmark injuries of osteoporosis, a condition that predominantly affects older adults. Women begin to experience osteoporotic fractures typically after 65 years of age due to earlier onset of bone loss, while men—whose higher peak bone mass offers temporary protection—often sustain such fractures later, commonly after age 80.^18^ These age thresholds directly correspond to the populations in our study who were least likely to receive prehospital analgesia and who experienced the longest delays in pain treatment upon ED arrival, despite representing the majority of fracture cases.

Age-related delays in pain management may stem from concerns about opioid-induced delirium in older adults, as opioids are associated with an approximately 2.5-fold increased likelihood of delirium in geriatric patients.^19^–^20^ However, decisions about pain medication should consider the clinical setting and involve a thorough, risk-based assessment. While this risk warrants caution, it should not preclude the timely use of non-opioid analgesics or appropriately dosed opioids, particularly for severe pain. Other potential mechanisms for the observed differences include under-reporting or atypical reporting of pain by older adults; implicit or cognitive biases from clinicians; variations in EMS and ED protocols; and operational constraints such as ED crowding, boarding, and staffing. Critically, none of these mediators were measured in our dataset, and they should, therefore, be viewed as hypothesisgenerating only.

The disparity in prehospital analgesia administration by age also raises concerns. Adults 18–64 years of age were significantly more likely to receive pain medications en route to the ED compared to older adults. Among geriatric patients, those ≥ 85 years of age were particularly vulnerable to undertreatment, despite presenting with similar or greater pain scores. Although many institutions, including ours, have protocols to guide analgesia administration based on pain scores, these systems may not sufficiently account for generational differences in pain expression. Older adults may under-report their pain compared to younger patients, which could unintentionally contribute to undertreatment.

### Implications for Practice and Research

This study adds to the evidence of age- and sex-based disparities in acute pain management. Future work should more precisely identify the drivers of these disparities. It should also prospectively evaluate solutions such as the following: protocolized, geriatric-aware multimodal pathways (opioid-sparing and regional anesthesia options); standardized assessment with mandated reassessment and patient-reported outcomes; clinician-focused strategies (education on pain in older adults, bias-aware training, audit-and-feedback); decision support/order sets that default to early non-opioid therapy; EMS standing-order refinement; and system-level actions addressing crowding, staffing, and documentation accuracy to determine whether these approaches shorten time to effective analgesia without increasing harm.

## LIMITATIONS

While this study has certain limitations, it also presents valuable opportunities for further research. The retrospective design, while limiting causal inferences, provides a strong foundation for identifying key disparities in pain management. Conducted at a single Level I trauma center, the findings offer important insights that future studies can build upon to assess generalizability across diverse settings. The analysis of prehospital analgesia highlights the need to explore factors such as clinician experience, clinician sex, and implicit bias in future investigations. Additionally, refining biologic sex categorization to better capture gender identity and incorporating more nuanced age stratification can enhance our understanding of patient experiences. Expanding the scope beyond femur and humerus fractures to include other injuries could further enrich findings. While this study primarily focuses on pharmacologic pain management, integrating non-pharmacologic strategies into future research may provide a more comprehensive view of pain treatment approaches.

## CONCLUSION

Age- and sex-based disparities in pain management for long bone fractures persist across both prehospital and ED settings. Despite the presence of robust pain protocols, older adults—particularly those ≥ 85 years of age—remain at increased risk for delayed or absent analgesia, although sex-based disparities appear to be narrowing. Because femur and humerus fractures are hallmark injuries of osteoporosis, affecting both older women and men, our focus on the intersection of age and sex across the entire care trajectory reflects a need to improve pain management for this vulnerable and growing patient population. Addressing these disparities requires both system-level changes, such as protocol refinement and training, and clinical-level improvements in clinician assessment and decision-making.

## Figures and Tables

**Table 1:** Patient demographics and emergency department visit characteristics for long bone fractures as part of a retrospective cohort study examining age and sex disparities in prehospital and emergency department pain management.

	Adults, 18–64 (n = 139)	Older Adults, ≥ 65 (n = 414)	All Patients (N = 553)
Patient demographics			
Age, years			
Median (Q1, Q3)	56 (38, 60)	81 (73, 87)	76 (64, 85)
Sex, n (%)			
Female	65 (46.8%)	284 (68.6%)	349 (63.1%)
Male	72 (51.8%)	128 (30.9%)	200 (36.2%)
Unknown/Did not disclose	2 (1.4%)	2 (0.5%)	4 (0.7%)
Fracture location, n (%)			
Femur	70 (50.4%)	315 (76.1%)	385 (69.6%)
Humerus	69 (49.6%)	99 (23.9%)	168 (30.4%)
Means of arrival, n (%)			
EMS arrival	91 (65.5%)	338 (81.6%)	429 (77.6%)
Non-EMS arrival	48 (34.5%)	76 (18.4%)	124 (22.4%)
Triage ESI, n (%)			
Level 1–2	34 (24.5%)	42 (10.1%)	76 (13.7%)
Level 3–4	104 (74.8%)	371 (89.6%)	475 (85.9%)
Unspecified acuity	1 (0.7%)	1 (0.2%)	2 (0.4%)
Treatment characteristics			
Time in treatment, minutes			
Median (Q1, Q3)	225 (148, 338)	270(198, 356)	262 (183, 355)
ED length of stay, minutes			
Median (Q1, Q3)	292 (180, 381)	303 (227, 398)	301 (216, 397)
Any analgesia, n (%)			
No	26 (18.7%)	90 (21.7%)	116 (21.0%)
Yes	113 (81.3%)	324 (78.3%)	437 (79.0%)
Any opioid, n (%)			
No	40 (28.8%)	138 (33.3%)	178 (32.2%)
Yes	99 (71.2%)	276 (66.7%)	375 (76.4%)
Arrival to pain medication, minutes			
Median (Q1, Q3)	44 (19, 88)	81 (41, 144)	72 (31, 132)
Clinician to pain medication, minutes			
Median (Q1, Q3)	19 (5, 48)	46 (17, 96)	37 (14, 83)

*ED*, emergency department; *EMS*, emergency medicine services; *ESI*, Emergency Severity Index; *Q*, quartile.

**Table 2:** Summary of pain medication administered for patients presenting to the emergency department with femur or humerus fractures.

EMS Arrival				

	All Patients	No Prehospital Analgesia	Prehospital Analgesia	Non-EMS Arrival
Received any analgesia, n (%)				
Age group, n (%)				
Adults, 18–64	113 / 139 (81.3%)	27 / 34 (79.4%)	47 / 57 (82.5%)	39/ 48 (81.3%)
Older adults, 65+	324 / 414 (78.3%)	142 / 192 (74.0%)	122 / 146 (83.6%)	60 / 76 (78.9%)
Sex, n (%)				
Male	153 / 200 (76.5%)	64 / 87 (73.6%)	57 / 73 (78.1%)	37 / 47 (78.7%)
Female	281 / 349 (80.5%)	103 / 137 (75.2%)	112 / 130 (86.2%)	68 / 85 (80.0%)
Time from ED arrival to analgesia, minutes				
Age group, median (Q1, Q3)				
Adults, 18–64	44 (19, 88)	47 (19, 73)	30 (15, 66)	70 (25, 149)
Older adults, ≥ 65	81 (41, 144)	85 (50, 149)	68 (41, 124)	101 (30, 171)
Sex, median (Q1, Q3)				
Male	57 (24, 118)	81 (42, 125)	30 (16, 59)	96 (35, 166)
Female	76 (41, 135)	83 (44, 139)	72 (44, 121)	86 (23, 154)

*ED*, emergency department; *EMS*, emergency medicine services; *Q*, quartile

**Table 3:** Comparison of time from emergency department arrival to first pain medication for long bone fractures by patient age and sex.

	Patients, n (%)	Arrival to first pain med in the ED, minutes	Adjusted percent difference (95% CI)	*P*-value
All Patients^1^				
Age Group				
Adults, 18–64	113 (25.9%)	44 (19, 88)	Reference	---
Older adults, > 65	324 (74.1%)	81 (41, 144)	+54.9% (+50.9%, +59.1%)	< .001
Sex				
Male	153 (35.3%)	57 (24, 118)	Reference	---
Female	281 (64.7%)	76 (41, 135)	+12.9% (+10.6%, +15.3%)	< .001
EMS Arrival with No Prehospital Analgesia^2^				
Age Group				
Adults, 18–64	27 (16.0%)	47 (19, 73)	Reference	---
Older adults, > 65	142 (84.0%)	85 (50, 149)	+73.0% (+63.7%, +82.9%)	< .001
Patient Sex				
Male	64 (38.3%)	81 (42, 125)	Reference	---
Female	103 (61.7%)	83 (44, 139)	+6.1% (−5.0%, +18.5%)	.29
EMS Arrival with Prehospital Analgesia^2^				
Age Group				
Adults, 18–64	47 (27.8%)	30 (15, 66)	Reference	—
Older adults, > 65	122 (72.2%)	68 (41, 124)	+77.2% (+68.8%, +86.0%)	< .001
Patient Sex				
Male	57 (33.7%)	30 (16, 59)	Reference	—
Female	112 (66.3%)	72 (44, 121)	+43.0% (+37.2%, +49.0%)	< 0.01
Non-EMS Arrival^2^				
Age Group				
Adults, 18–64	39 (39.4%)	70 (25, 149)	Reference	---
Older adults, 65+	60 (60.6%)	101 (30, 171)	+32.8% (+15.7%, +52.4%)	< 0.001
Patient Sex				
Male	37 (25.2%)	96 (35, 166)	Reference	---
Female	68 (64.8%)	86 (23, 154)	−6.8% (−20.4%, +9.0%)	0.38

1 Percent difference was adjusted for triage ESI, first pain score in the ED, means of arrival, and prehospital analgesia.

2 Percent difference was adjusted for triage ESI and first pain score in the ED.

*ED*, emergency department; *EMS*, emergency medicine services; *ESI*, Emergency Severity Index.
